# Chemical and thermal stabilization of CotA laccase via a novel one-step expression and immobilization in muNS-Mi nanospheres

**DOI:** 10.1038/s41598-021-82468-x

**Published:** 2021-02-02

**Authors:** Tomás Pose-Boirazian, Gemma Eibes, Natalia Barreiro-Piñeiro, Cristina Díaz-Jullien, Juan M. Lema, Jose Martínez-Costas

**Affiliations:** 1 Centro Singular de Investigación en Química Biolóxica e Materiais Moleculares (CiQUS), Departamento de Bioquímica y Biología Molecular, Universidade de Santiago de Compostela, 15782 Santiago de Compostela, Spain; 2 CRETUS Institute, Dept. of Chemical Engineering, Universidade de Santiago de Compostela, 15782 Santiago de Compostela, Spain; 3 Centro de Investigación en Bioloxía (CiBUS), Departamento de Bioquímica y Biología Molecular, Universidade de Santiago de Compostela, 15782 Santiago de Compostela, Spain

**Keywords:** Biocatalysis, Enzymes, Proteins, Biotechnology, Biomaterials, Expression systems, Nanobiotechnology

## Abstract

A methodology that programs eukaryotic or bacterial cells to encapsulate proteins of any kind inside micro/nanospheres formed by muNS-Mi viral protein was developed in our laboratory. In the present study such “in cellulo” encapsulation technology is utilized for immobilizing a protein with an enzymatic activity of industrial interest, CotA laccase. The encapsulation facilitates its purification, resulting in a cost-effective, one-step way of producing immobilized enzymes for industrial use. In addition to the ability to be recycled without activity loss, the encapsulated protein showed an increased pH working range and high resistance to chemical inactivation. Also, its activity was almost unaffected after 30 min incubation at 90 °C and 15 min at the almost-boiling temperature of 95 °C. Furthermore, the encapsulated laccase was able to efficiently decolorate the recalcitrant dye RB19 at room temperature.

## Introduction

Enzyme immobilization is a useful technology to increase the thermal and chemical stability required for many biotechnological applications^1^. Among many different options, microparticles have been gathering increasing attention over the years for their multiple applications in very different areas, ranging from industrial production to therapeutic treatment or diagnosis^2^. However, the productions costs and the stability of the microspheres are the main factors limiting their application at higher scale.

In previous works, a methodology that programs cells to construct spheres made by a viral protein called muNS-Mi (microspheres or MS in mammalian or insect cells, 1 to 4 μm; nanospheres in bacteria, or NS, 400 nm), and to load those spheres with any protein of interest, providing that it has been previously tagged with a sequence called intercoil (IC) has been developed. The so-called IC-Tagging methodology is based on the ability of the muNS protein from avian reovirus S1133 to form the matrix of the viroplasms that S1133 forms inside infected cells^3^. While protein muNS forms irregular, but ordered, protein inclusions when expressed alone, the truncated version muNS-Mi forms regular protein spheres^4^. Such spheres are able to recruit in living cells any foreign protein that bears the muNS-Mi domain named IC, either on the N or C terminus^5^. They are easily purified only by mechanical means in a cost-effective way, and any IC-tagged protein that has been co-expressed with them will be integrated into and co-purified with the MS or NS. It was also shown that several different IC-tagged proteins can be simultaneously loaded into the MS. Purified MS can serve as subunit vaccines when loaded with specific IC-tagged viral antigens without the need of added adjuvants^6–9^. It has also been demonstrated the utility of this methodology for the detection of protein–protein interactions in living cells^10^. Integration into MS and NS allows the expression of proteins that are otherwise very difficult to express, and a version of the method that is functional through the secretory pathway allows the expression and purification of glycoproteins loaded in MS^11^. Complex interactions were also shown to happen between protein monomers while integrated into these structures^12^.

With our method, both the expression and the micro/nano-encapsulation are performed simultaneously by the cell, avoiding the need of protein purification and the subsequent coupling to a surface/particle. For that, no particular additives need to be incorporated to the normal growth media, and particle formation facilitates the encapsulated protein purification by only mechanical means, overall reducing the cost of the entire production process. As our methodology allows the cost-effective production and purification of encapsulated proteins, we wanted to explore the ability of the NS as enzyme carriers to promote both, enzyme reusability and stabilization of the integrated enzyme that might increase the applicability of the IC-tagging method for protein production.

As a model enzyme the laccase-like enzyme codified by the *Bacillus subtilis* CotA gene was chosen. Laccases (EC 1.10.3.2) are the largest subgroup of the protein superfamily of multi-copper oxidases (MCOs) and catalyze the one electron oxidation of a broad range of substrates using molecular oxygen as the final electron acceptor. Their broad substrate spectrum and their ease of use have generated a high interest, particularly within the wood, biofuel, paper, textile, fine chemicals and food industrial sectors^13^. Different laccases from plant, fungal and bacterial origin have been identified and characterized. The remarkable characteristics of bacterial laccases from the industrial point of view, such as a broad range of temperature and pH with high stability against various inhibitory agents, have boosted their use and application in recent years^14^. CotA laccase is present in the coat of the *B. subtilis* endospore and has been previously characterized biochemically^15^.

In this study we produced and purified NS loaded with CotA and characterized the activity of the nano-encapsulated enzyme. The immobilized enzyme can be efficiently recycled and is able to decolorize the RB19 recalcitrant dye.

## Results

### Expression and purification of encapsulated CotA laccase

**Figure 1:**
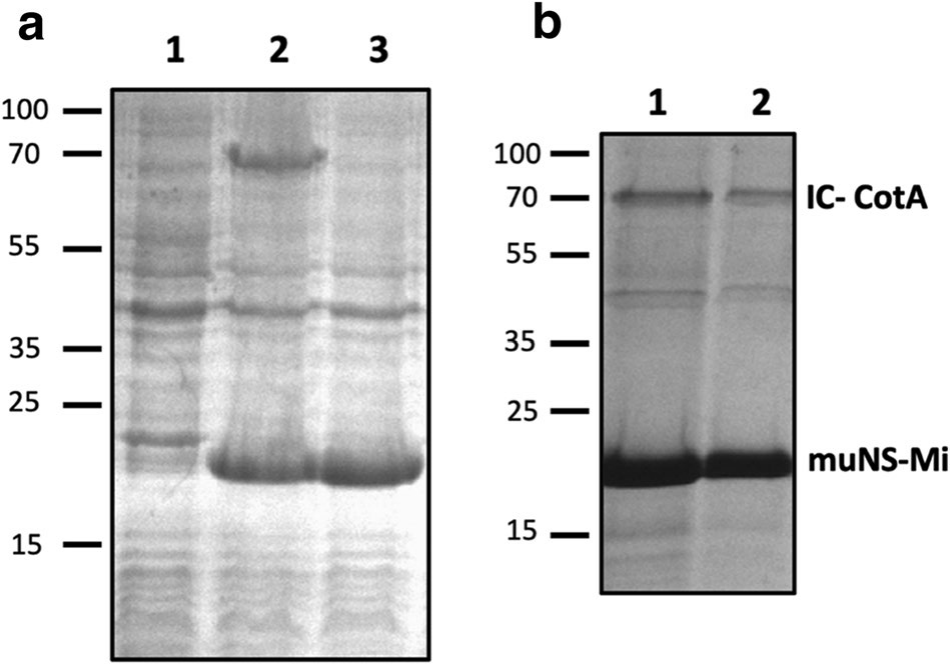
Purification of NS-CotA. (**a**) Coomassie-blue stained, PAGE analysis of extracts from non-induced (1), or IPTG-induced (2) *E. coli* transformed with dual expression plasmid muNS-Mi-CotA-pDuet1. Extracts from *E. coli* transformed with plasmid muNS-Mi-pDuet1 and induced with IPTG are shown in 3. (**b**) Protein content of the purified NS-CotA produced under standard (1) or microaerobic (2) conditions, analyzed by SDS-PAGE and Coomassie staining.

The coding sequence of the CotA laccase was obtained by PCR amplification from the purified genome of *B. subtilis*. In order to produce the enzyme encapsulated into muNS-Mi nanospheres, the IC-Tag sequence was added to the C-terminus of the CotA gene to generate the fusion construct CotA-IC. Additionally, to be completely sure of the simultaneous intracellular expression of muNS-Mi and CotA, the IC-tagged sequence of the *B. subtilis* enzyme was cloned into the second polylinker of plasmid muNS-Mi-pDuet1, a dual expression plasmid bearing the muNS-Mi sequence in the first polylinker, a construct that was previously produced in our laboratory. BL21 cells transformed with the recombinant dual plasmid and whose expression was induced with IPTG, showed no band at all corresponding to the calculated molecular weight of CotA-IC (not shown). Alternatively, the Rossetta strain of *Escherichia coli* was used to overcome a possible difference in codon usage between *B. subtilis* and *E. coli*. After IPTG induction of the transformed Rossetta cells, total cell extracts were obtained and analyzed by SDS-PAGE, showing the presence of two prominent bands that were not present in extracts from uninduced cells (Fig. 1a, compare lanes 1 and 2). The faster migrating band corresponds to muNS-Mi and is also present in extracts from Rossetta cells transformed with the muNS-Mi-pDuet plasmid (Fig. 1a, lane 3), while the size of the slower migrating protein is in agreement with the size (64 kDa) of the IC-tagged CotA laccase from *B. subtilis*. The yield of purified laccase in the NS was between 30 and 50 mg/L of culture.

Next, the nanospheres made by muNS-Mi inside induced bacteria were purified using a simple and inexpensive protocol previously developed in our laboratory (see Methods). The presence and size of the NS were analyzed by DLS, showing a monodisperse population of particles of around 400 nm in diameter (not shown). The protein composition of the purified particles was analyzed by SDS-PAGE showing the presence of 2 proteins with apparent molecular weights corresponding to muNS-Mi and CotA-IC (Fig. 1b).

### Activity of nanoencapsulated IC-CotA

After the NS purification, we wanted to test if the encapsulated protein kept its enzymatic activity by measuring the transformation of ABTS as substrate. Our initial attempts produced no detectable activity after incubation at the published CotA optimal pH. Those results were expected as it was previously published that incorporation of the essential copper atoms to the active center of CotA is dependent on oxygen, and incubation in microaerobic conditions and addition of copper to the incubation medium (see Methods) are necessary for obtaining fully copper loaded enzyme molecules^16,17^. However, laccase-containing MS produced in the baculovirus system showed some activity in the absence of any particular incubation conditions or additives (our unpublished results). Thus, the production of the nanoencapsulated enzyme under standard conditions served first, to confirm that the predictable stabilization effect of the encapsulation makes no difference on the requirements for obtaining an active enzyme in bacteria, and second, as a control on the possible effect that the muNS-Mi protein might have on the transformation of the substrate ABTS used for the laccase activity determination. Next, a new batch was obtained under microaerobic conditions in LB medium containing 0.25 mM CuCl_2_. Similar results to those shown in Fig. 1 were obtained in terms of IPTG-induction of protein expression. However, a decrease of 20% in total protein was obtained under microaerobic conditions that produced a similar decrease in total purified NS (Fig. 1b, compare lane 1 with lane 2, quantified as indicated in Materials and Methods). However, estimation of bacterial growth by absorbance measurement (OD 600), showed that a similar decrease in cell growth under microaerobic vesus standard conditions. Thus, the observed difference in protein production is due to a difference in cell number, demonstrating that the presence of Cu and the microaerobic culture conditions have no detrimental effects on the expression of either muNS-Mi or CotA-IC. In addition, this Cu-loaded CotA did produce the appearance of a colored product after incubation with ABTS, indicating the presence of oxidase activity on the purified NS. The specific activity of NS-CotA measured at standard conditions was 39 mU/mg NS. Next, the activity of the encapsulated CotA was characterized and compared with a commercially available enzyme in its free soluble form.

### Effect of pH on CotA activity and stability

**Figure 2:**
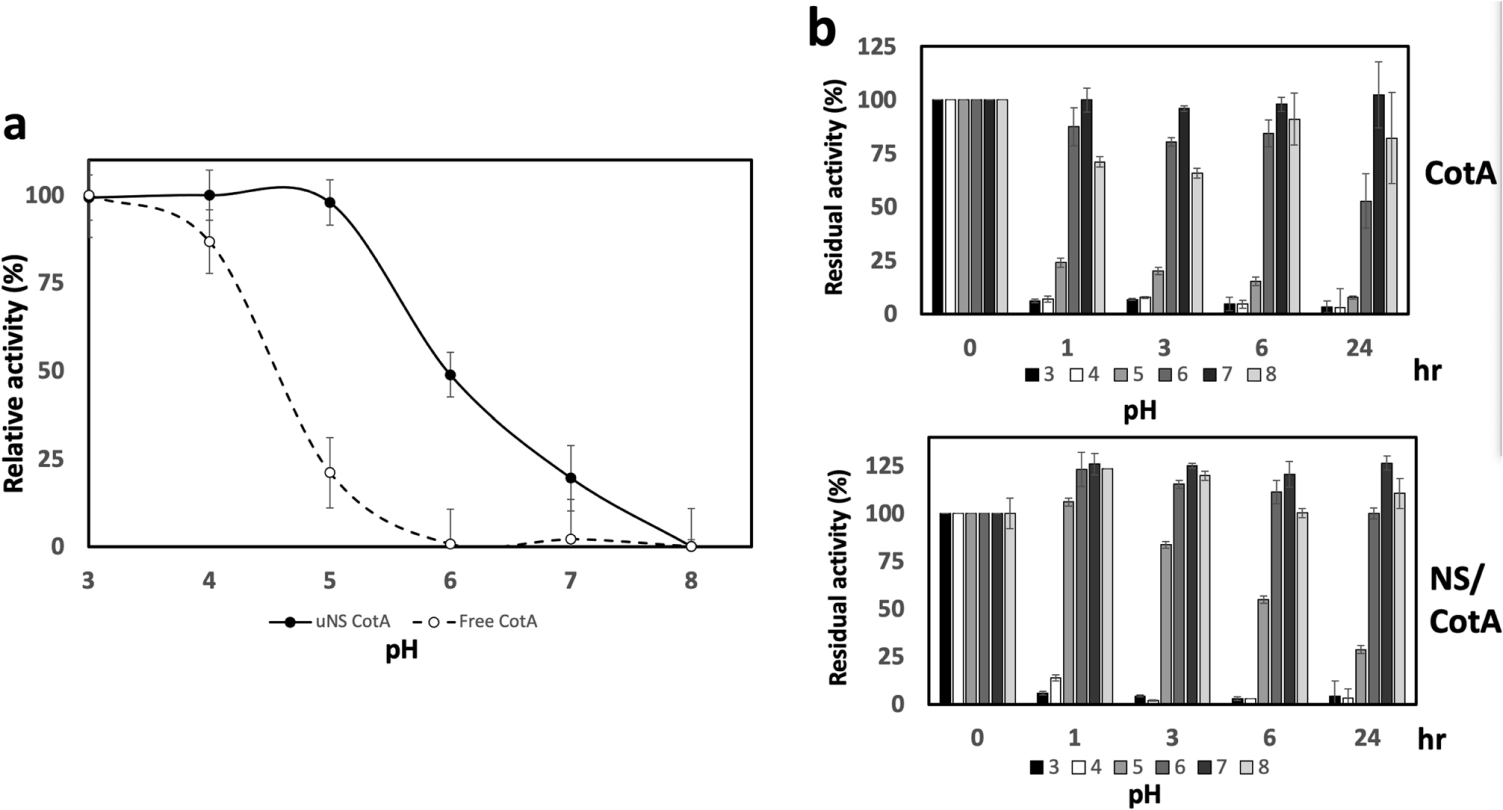
Comparison of pH activity and stability between free CotA and NS-CotA. (**a**) Optimum pH. The graphic shows the ABTS-oxidizing activity of free (open circles) and immobilized (closed circles) CotA at pH ranging from 3 to 8. (**b**) pH stability. Residual activity (%) of free CotA and NS-CotA after incubation at different pH values (3 to 8).

The pH-activity profile of the NS-contained CotA laccase was first compared to the one obtained with the free enzyme. The free CotA presented an optimum pH activity, as previously reported^18^, around pH 3.0 and 4.0 with a rapid decline of relative activity at pH 5 and almost undetectable at pH 6 (Fig. 2a, open circles). However, the immobilized version presented a significative expansion of the optimum pH, covering a range between pH 3.0 and pH 5.0 (Fig. 2a, closed circles). In addition, NS-contained CotA maintained a 50% ABTS-oxidizing activity at pH 6.0 and 20% at pH 7.0 while the free enzyme was nearly inactive at those pH values. As the incorporation into NS seems to stabilize the enzymatic activity of CotA, the resistance to incubation during 24 h of free and NS-contained CotA in different pH ranging from 3 to 8 was compared. The results showed that at pH 3.0 and 4.0 both enzymes had a similar behavior (Fig. 2b, compare the white and black bars from CotA vs NS-CotA). However, integration into NS improved CotA stability over the more neutral pH range. Thus, while free CotA laccase showed only 24% relative activity after 1 h incubation at pH 5.0 and decreased to a 8% activity after 24 h, the immobilized version did not experience a loss in activity during the first hour at the same pH, retained a 86% activity after 3 h and after 24 h still preserved a 28% activity. An increased stability of NS-CotA over the free enzyme was also evident at pH 6, while incubation at pH 7 and 8 did not apparently decrease the activity of any of the two enzymes.

### Stability against inactivating agents

**Figure 3:**
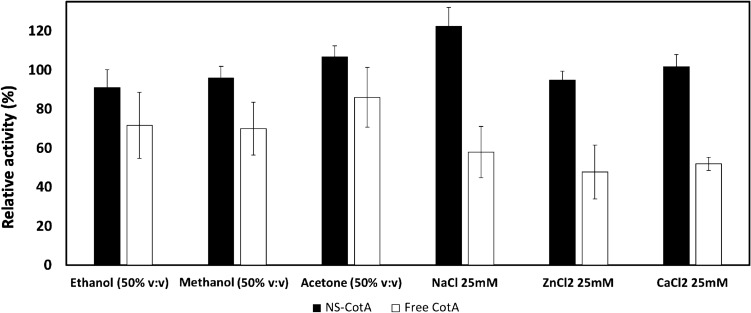
Residual activity (%) of free CotA (white bars) and immobilized CotA (NS-CotA, black bars) after 1 h incubation with different inactivating agents shown in the figure at room temperature.

The next step was checking if the nanoencapsulation also confers stability against chemical inactivation. For that, free and NS-contained CotA were incubated for 1 h at room temperature with the reagents shown in Fig. 3 and then their activity was measured under standard conditions. Three different chloride salts were evaluated to simultaneously check a possible effect of the metal ions on laccase activity^19^. The results clearly show an increased stability of the NS-CotA over the free enzyme. Thus, the NS-CotA showed an extraordinarily tolerance to the presence of both organic solvents: 91% stability in 50% ethanol (v/v), a 95% stability in 50% methanol (v/v) and a 100% stability in 50% acetone (v/v) as well as for other chemical inactivators: a 122% stability against 25 mM NaCl, a 94% stability against 25 mM ZnCl_2_ and 100% stability in the presence of 25 mM CaCl_2_.

### Optimum temperature and thermostability

**Figure 4:**
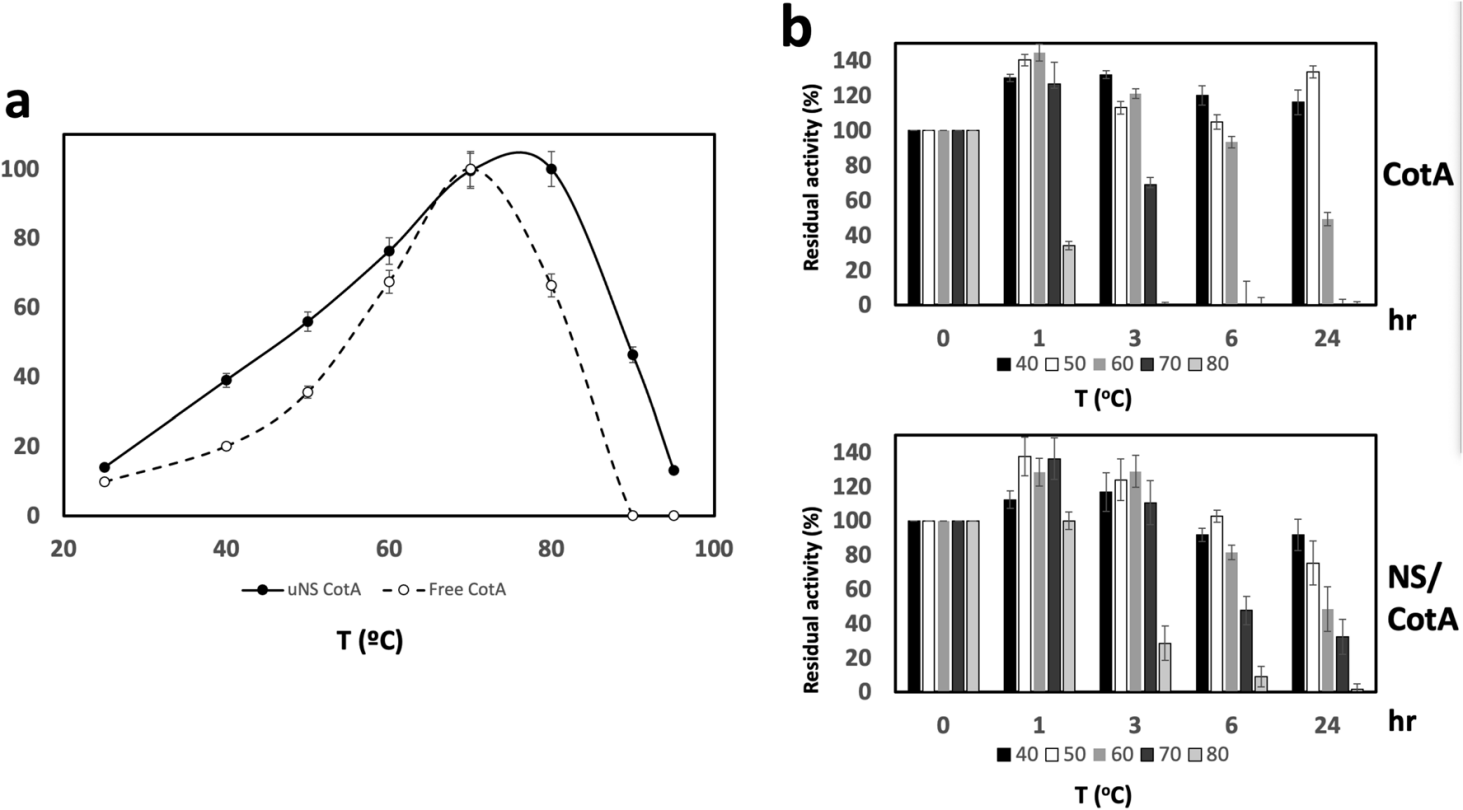
Influence of temperature on CotA activity. (**a**) Effect of temperature on ABTS-oxidizing activity of free (open circles) and immobilized CotA (closed circles). (**b**) Residual activity (%) of immobilized CotA (NS/CotA) and free CotA after incubation during the indicated times (h) and different temperature values indicated by different colour bars.

To determine and compare the temperature range of action of both CotA versions, standard reactions were performed with both enzymes at different temperatures. The free CotA (Fig. 4a, open circles) was slightly more active at 70 °C, but the nanoencapsulation increased the optimum temperature by 10 °C to the 80 °C (Fig. 4a, filled circles). Furthermore, NS-CotA presented an increased relative activity in the entire range of temperatures from 25 to 95 °C, being this increase more pronounced for the higher temperatures. Thus, for example, the free CotA became completely inactive at 90 °C but NS-CotA showed a 50% relative activity at that temperature. Furthermore, NS-CotA showed a similar relative activity at 25 °C and at the striking temperature of 95 °C.

In view of those results the thermostability of both enzymes was compared. For that, free and NS-contained CotA were incubated at pH 7 for different incubation times (Fig. 4b) at selected temperatures ranging from 40 to 80 °C. NS-CotA (Fig. 4b, upper panel) presented superior half-lives and lower inactivation rates when compared to the free enzyme (Fig. 4b, lower panel), being more accentuated at the higher temperatures. After 1 h of incubation both versions retained full activity from 40 to 70 °C but at 80 °C the free CotA showed only a 34% relative activity (Fig. 4b, lower panel) while NS-CotA remained totally active (Fig. 4b, lower panel). A similar behavior took place at 6 h where both versions remained totally active from 40 to 60 °C. At the same incubation time and 70 °C and 80 °C, however, the free CotA was completely inactivated while the immobilized CotA retained almost 50% of the relative activity at 70 °C and a 10% activity at 80 °C.

**Figure 5:**
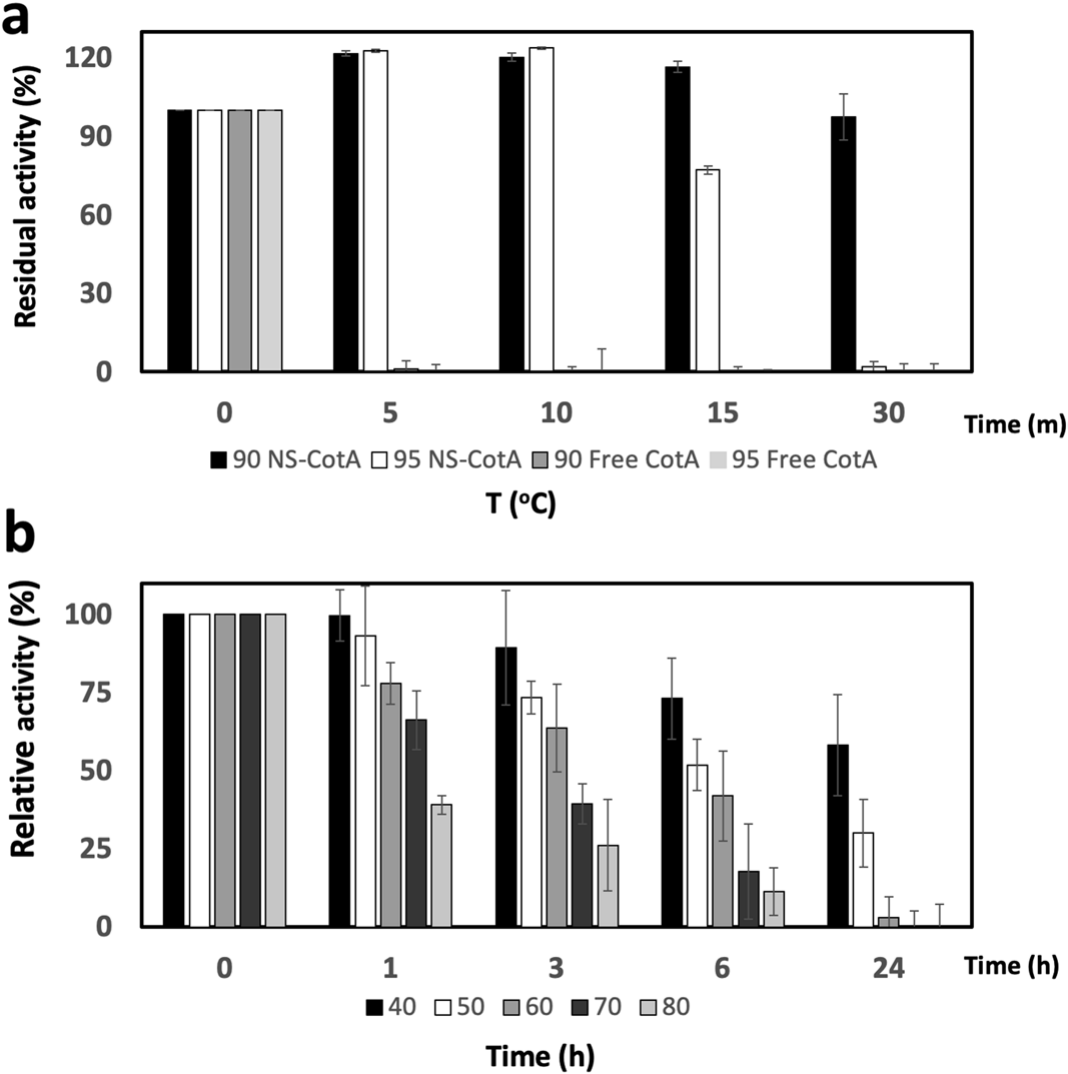
Stability of CotA activity at high temperatures. (**a**) Residual activity (%) of free CotA (grey bars) or NS-CotA (white and black bars) after incubation at the times indicated (min) and at 90 (black and dark grey bars) and 95 °C (white and light grey bars). (**b**) Residual activity (%) of free IC-CotA after incubation during the indicated times (h) and different temperature values indicated by different colour bars.

With those results in mind, we decided to compare the behavior of both enzymes at even higher temperatures (90 and 95 °C) for 30 min and checking the activity at different time points. The stability at these temperatures is strikingly different (Fig. 5). Thus, the free enzyme (Fig. 5, grey bars) was completely inactivated at 5 min at both temperatures, while NS-CotA activity (Fig. 5, black and white bars) was not affected after 10 min of incubation at either temperature. At 90 °C the activity of NS-CotA was also unaffected after 30 min of incubation and still retained 77% of its activity after 15 min of incubation at 95 °C. Incubation at 95 °C for 30 min were the only conditions found to eliminate the activity of the extremely heat-stable NS-contained enzyme.

Although both, the free and the NS-contained enzymes are CotA from *B. subtilis*, we are adding the IC-Tag to one end of the immobilized, while the free CotA is a purified version produced and provided by a company (see “Materials and Methods”). Thus, to discern if the high stability shown by the nanoencapsulated version is due to encapsulation or just to the presence of the IC-tag, a heat-stability assay similar to the one shown in Fig. 4b was performed by using extracts from bacteria expressing either NS-IC-CotA, or free IC-CotA. While the results obtained for the encapsulated enzyme matched exactly those on Fig. 4b as expected, the free IC-tagged CotA showed a diminished resistance to heat inactivation than the purified, commercially available laccase that we used as a control over this study, clearly demonstrating that the stabilization effect was caused by the encapsulation inside NS.

### Reusability of NS-CotA and ability to decolorate organic dyes

**Figure 6:**
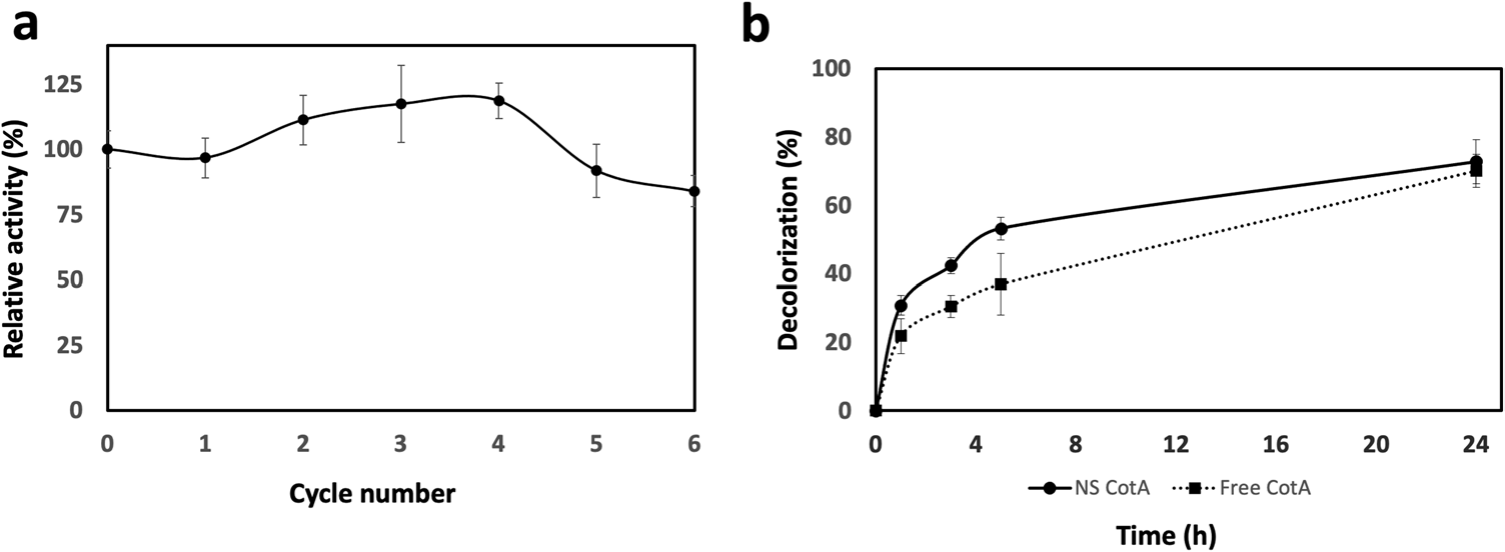
(**a**) Residual activity (%) of NS-CotA during 6 cycles of 5 mM ABTS oxidation. (**b**) Decolorization of RB19 by NS-CotA (closed circles) and free CotA (closed squares) at 60 °C and pH 5.0.

Having demonstrated the increased stability of the encapsulated enzyme, the next step was to proof if such encapsulation allowed its recovery and reuse in several reaction cycles, as this is one of the most critical aspects for potential industrial applications. For that, the activity of NS-CotA was measured as above and then the NS recovered by centrifugation. The pelleted NS were then resuspended again in reaction buffer containing ABTS and the activity measured. As can be seen in Fig. 6a, CotA activity was entirely conserved during 4 cycles, and after 6 cycles of use showed only a 20% loss of relative activity, a figure that matches exactly the percentage of protein lost after all the centrifugation steps used for the recovery.

The recalcitrant RB19 dye was used as a model compound to test the decolorization efficiency of the immobilized CotA at pH 5 and 60 °C, in a final volume of 1 ml reaction mixture containing 100 U/L of immobilized enzyme. A negative control with inactivated enzyme (not shown) did not show any significative RB19 decolorization, thus we can associate the enzymatic activity as the responsible for the removal of the dye. The reaction was monitored for periods of 1, 3, 6 and 24 h to observe the progression (Fig. 6b). The results showed that NS-CotA, in absence of any mediator, catalyzed 72.8% of the RB19 decolorization after 24 h, showing a similar outcome to that of the free enzyme. The results also showed that most of the dye elimination took place during the first hours of reaction. Interestingly, the immobilized enzyme showed higher initial reaction rates compared to that of the free enzyme (53.2% and 37% decolorization after 5 h for immobilized and free enzyme, respectively). Future studies are needed to optimize dye removal, considering variables such as temperature, pH, enzyme dosage or addition of a mediator.

## Materials and methods

### Reagents and enzymes

Glutaraldehyde (70%), 2,2′-azino-bis (3-ethylbenzothiazoline-6-sulfonic acid) (ABTS), sodium dodecyl sulfate (SDS), NaCl, ZnCl_2_, CaCl_2_, CuCl_2_ etc., were purchased from Sigma-Aldrich. Reactive Blue 19 (RB19), also known as Remazol Brilliant Blue or disodium salt of 1-amino-2-sulfo-4-(3-sulfoxy-ethyl-sulfo-phenyl-1-ylamino)-5,10-anthraquinone was obtained from Sigma-Aldrich (St. Louis, MI, USA). Organic solvents such as methanol, ethanol, acetone were obtained from Merck. All chemicals were of analytical grade (or the highest purity available). Bacterial CotA laccase (L111) was kindly provided by Metgen (Oy, Kaarina, Findland).

### Bacterial strains

*E. coli* strain XL1-Blue (Stratagene, La Jolla, California) was used to grow and purify plasmids. BL21-CodonPlus-RP (DE3) (Agilent Technologies) and Rosetta (DE3) Competent Cells—(Novagen) were used for protein expression.

### Plasmid construction

Bacterial muNS-Mi: plasmid pET Duet-Mi that expresses muNS-Mi in bacteria has been previously described^11^.

Dual-muNS-Mi/IC-CotA: First, a plasmid containing the IC-tag sequence followed by a Factor Xa cleavage site was created. For that, plasmid pCINeo-muNS1 was subjected to PCR amplification with the following primers: the forward primer was 5′-GCGCCCAAGCTTATCATGGAAGATCACTTGTTGG-3′ (HindIII site is single underlined and ATG initiator is double underlined) and reverse primer was 5′-GCGGGTACCCCTTCCCTCGATCGCTTCC-3′ (KpnI site is single underlined and Xa factor is double underlined). The PCR product was digested and cloned into the plasmid pcDNA 3.1 Zeo + to generate the plasmid pcDNA3.1Zeo-IC-Xa. Then the sequence of CotA was obtained by PCR amplification from the genomic DNA of *B. subtilis*. The forward primer was 5′-CGCTGCAGTATG ACACTTGAAAATTT-3′ (PstI site is single underlined) and reverse primer was 5′-CGCGCGGCCGC TTATTTATGGGGATCAGTT-3′ (NotI site is single underlined and stop codon is double underlined). The PCR product was digested and cloned into the plasmid pcDNA3.1Zeo-IC-Xa to generate the plasmid pcDNA-IC-Xa-CotA. The whole construct was obtained by PCR amplification from pcDNA-IC-Xa-CotA by using the forward primer: 5′-GCGAGATCTATGGAAGATCACTTGTTGGCTTAT-3′ (BglII site is single underlined) and reverse primer: 5′-CGCCTCGAGTTATTTATGGGGATCAGTT-3′ (XhoI site is single underlined and stop codon is double underlined) and cloned into the MCS2 of the plasmid pET Duet-Mi to generate the pET Duet-Mi-IC-CotA.

### Expression and purification of nanoencapsulated CotA laccase

BL21 CodonPlus-RP (DE3) or Rosetta (DE3) competent bacteria were transformed with plasmid pET Duet-Mi-CotA and incubated at 37 °C with shaking to reach at OD600 ~ 0.4–0.6. *Standard conditions:* expression was induced with 1 mM IPTG and incubated during 3 h at 37 °C with shaking. *Microaerobic conditions:*^16,17^ The transformed bacteria were diluted with LB medium supplemented with 0.25 mM CuCl_2_ and incubated at 25 °C with shaking at 100 rpm for 5 h. After that, shaking was stopped and the bacteria were further incubated overnight. The NS were purified essentially as described:^11^ induced cultures were centrifuged at 3200Xg and the pellet washed twice in PBS. Then, the bacteria were resuspended in 1/10 volume of lysis buffer (0.25% Tween-20, 1 mM DTT, 200 mM NaCl, 20 mM Tris pH 7.5, 2 mM MgCl_2_) and frozen. The pellet was thawed and bacteria lysed by three passes through a French Press. Then, NS were centrifuged at 2700Xg, 5 min at 4 °C, washed once in RB + buffer (10 mM Hepes pH 7.9; 10 mM KCl; 5 mM MgCl_2_) containing 0.5% Triton X-100 once, washed fivefold in RB+, and finally resuspended in a small volume of RB. The diameter of the NS was measured by dynamic light scattering (DLS) analysis after diluting them 10 times and transferred to a standard disposable cuvette for size measurements. All experiments were done in triplicate at 25 °C on a Zetasizer Nano ZSP (Malvern). For quantification of protein yield, samples of the NS loaded with the enzyme were first incubated in SDS 10% to denature the proteins and disassemble the nanospheres. Total protein content was measured using the DC Protein Assay Kit (Bio-Rad), following the manufacturer’s instructions. Then, for routine measurements, a direct relation between total protein content and absorbance was established by measuring previously quantified samples on a spectrophotometer UV–VIS Nanodrop 1. To measure the laccase content, the protein ratio corresponding to each of the nanosphere components was quantified by densitometric analysis of a polyacrylamide gel containing serial dilutions of the samples. Thus, the enzyme concentration was calculated from the total protein content. The results obtained were confirmed by comparing serial dilution of the NS samples with serial dilutions of a solution of BSA (68 kDa) of known concentration.

### Laccase activity assays

Kinetic studies were carried out in triplicate in a reaction mixture containing 5 mM ABTS in citrate–phosphate buffer at the pH and temperature indicated in each assay. The oxidation of ABTS was followed by measuring the absorbance at 420 nm (ε = 36,000 M^−1^ cm^−1^) at 25 °C in a Tecan Infinite 200 Pro microplate reader. One unit (U) of activity was defined as the amount of enzyme forming 1 µmol of ABTS^+**.**^ radical per minute. All spectrophotometric measurements were carried out using a Jasco V-770 UV–Visible/NIR Spectrophotometer. Laccase activity was expressed in U/L. All measurements were carried out in triplicate.

### Optimization of laccase transformation assays

The pH optimum. This was investigated using 50 mM ABTS in a 0.1 M citrate–phosphate buffers of pH ranging from 3 to 8. The relative activity was calculated as the ratio between the activity at each pH and the maximum attained, set as 100%. The effect of the pH on the enzyme stability was studied by incubating the enzyme in 0.1 M citrate–phosphate buffer (pH 3–8) at room temperature (24 ± 2 °C) during 1,3,6 and 24 h. Samples were transferred to standard reaction mixtures in order to determine the laccase activity under standard conditions. The residual activity was calculated referred to the value of the initial activity at each pH. All measurements were performed in triplicate.

Optimum temperature and thermostability. The heat stability of NS-CotA was compared to a commercially available *B. subtilis* CotA laccase. The effect of temperature (25–95 °C) was determined by measuring activity at the corresponding temperature under standard conditions^16^. The relative activity was calculated as the ratio between the activity at each temperature and the maximum attained. Thermal stability was determined by incubating 160 U/L of the enzymes in 0.1 M phosphate buffer (pH 7) at selected temperatures from 25 to 95 °C over a period of 24 h or the times indicated in each figure. Samples were then transferred to standard conditions to determine the relative activity.

### Stability of laccase

Stability against inactivating agents. 100 µl (10U) of enzyme were incubated in different solutions (50% (v/v) methanol, 50% (v/v) ethanol, 50% (v/v) acetone, 25 mM NaCl, 25 mM CaCl_2_ and 25 mM ZnCl_2_) in 5 mM citrate–phosphate buffer, pH 7 at room temperature. The residual activity was measured with ABTS under standard conditions after 1 h incubation.

Storage stability was investigated at 4 °C and at room temperature (24 ± 2 °C). Enzyme samples, with an initial activity of 160 U/L, were stored in a 0.1 M citrate–phosphate buffer solution (pH 7). The residual activities were measured after 1, 2 and 3 months by measuring the remaining CotA activity with 50 µM ABTS under standard conditions.

### Reusability of the NS-laccase

The reusability of the immobilized laccase was evaluated in the operation of consecutive cycles using ABTS as substrate under standard conditions and with an initial activity of 160 U/L. The NS-CotA was collected by centrifugation and washed twice, one with RB+ and one with RB−. Thereafter, the procedure was repeated with a fresh aliquot of substrate. The reference value of 100% was the activity of the immobilized enzyme in the initial cycle.

### Decolorization of RB19 by laccase

The removal percentage was determined by measuring the absorbance of the test samples by spectrophotometry at 593 nm. The enzymatic transformation of RB19 was carried out over a period of 24 h at pH 5 and 60 °C in a final volume of 1 ml reaction mixture containing 12 mg/L of the dye and 100 U/L of enzyme. Heat-inactivated laccase was used as negative control. After decolorization, the absorbance of the reaction mixtures was then measured at 593 nm with a Tecan Infinite 200 Pro microplate reader.

## Discussion

Enzymes are widely used in industry, particularly important in sectors such as leather processing and bioethanol production, but also present in the food industry, textile industry, cosmetics, medicinal products, detergent industry, pulp and paper application, etc. Current prospects of commercial enzymes and future demand are increasing dramatically^20^. However, the production of enzymes has been always a challenge. Its purification is known to be costly and time consuming, being chromatography the most applied technique. In fact, downstream processing can represent up to 80% of the production cost of manufacturing biomolecules^21^. As a result, the production of enzymes in many cases is not cost effective.

Another major challenge in industrial biocatalysis is the development of stable, robust, and reusable biocatalysts. This can generally be overcome by immobilization of enzymes. Although the idea of immobilizing enzymes for biocatalysts purposes is not new^1,22–24^, challenges remain for a stable, efficient, cost effective and simple method for the production of immobilized proteins. Covalent attachment methods are usually preferred, despite their complexity with few preparative steps, and they have to be carefully selected for not disturbing the activity of the immobilized enzyme^25^. Hence, the conventional strategy to obtain stable and reusable enzymes consist on the expression of the enzyme, its purification and the later immobilization, being the whole process a combination of different steps and involving larger amounts of enzyme than the eventually immobilized. Thus, a one-step method for both expression and immobilization would be highly desirable as far as it preserves activity, and this can be achieved using self-assembling in vivo techniques that allow the proper enzyme folding and post-translational modifications while integrated into the carrier. The purification stage of the immobilized enzyme proposed here, based on washing and centrifugations steps, is simple and inexpensive if compared with the multiple bioprocessing steps of enzymes purification, including chromatography and other polishing steps. Zhou et al. (2021)^26^ recently reviewed the multiple immobilization methods and immobilized carriers for laccases considering traditional, but also new materials. According to this paper, despite the advanced methods developed in recent years, there are still two main problems limiting the practical applications of immobilized laccase: the high cost and laccase activity decrease. The technology proposed in the present study attempts to overcome the two problems. The simple and inexpensive one-step expression and immobilization of laccase produces a ready-to use catalyst, showing high stability and reusability. Compared to conventional production of laccase, the difference of the present process is the simple purification. Since laccase is yet integrated in the nanocarrier, only washing and centrifugation steps are required. The complex protocols for enzyme purification plus immobilization are not required, avoiding the use of chemicals (sometimes hazardous) or carriers. Furthermore, it is avoided the activity loss suffered during most immobilization processes (particularly in covalent immobilization). This work proves that this technology is feasible to produce immobilized laccase. Further research to optimize the expression yields (only standard conditions were studied) and the scale-up would allow the economic study of this technology.

To test if our IC-Tagging methodology would result a good one-step enzyme immobilizing method, we chose the *B. subtilis* laccase, first because of its multiple applications in different fields as industry or biotechnology, but also because its complex globular structure comprising an active center that should contain four coordinated cupper atoms to be active. *Bacillus* laccase was produced at relevant concentrations by using the Rosetta strain of *E. coli*. The enzyme co-purified with muNS-Mi after lysis and centrifugation showing its integration into NS. Expression yields of 30–50 mg/L were observed under standard conditions, without performing any expression optimization studies. These are similar or even higher than values reported in previously works where optimization of CotA laccase production in *E. coli* was performed^27^. However, in spite of the laccase content, no activity was present in the NS when the expression was performed under standard conditions, in keeping with the reported observation that only after supplementing the culture medium with copper and carrying out the expression under microaerobic conditions, the enzyme would get loaded with the necessary copper ions for carrying out its activity.

The copper-loaded enzyme was then produced at similar yields than the inactive version, and its activity was characterized by measuring its ability to oxidize ABTS. The immobilized activity was similar to the values reported for different laccases encapsulated by biomimetic silica mineralization (39 U/g NS vs. 15.3–126.7 U/g support)^28^. We found that the NS-contained enzyme presented an increased activity over a wider pH range when compared to the free enzyme, what agrees with a stabilizing effect of the nanoencapsulation on the enzyme. Furthermore, the stability of the enzymatic activity against incubation at different pH values also showed a remarkable increase upon nanoencapsulation.

Identical stabilization effects were observed on incubation with different inactivating agents such as organic solvents and salts. Concentrations of different reagents that produced a deleterious effect on the activity of the free enzyme had no effect on the NS-contained laccase. The increased stability in organic solvents is particularly interesting for the industrial application of enzymes. The use of organic solvent systems instead of aqueous media for enzymatic reaction offers numerous advantages; however, organic solvents often inactivate enzymes^29^. On the other hand, halides are known inhibitors of laccases, which may limit their application for bioremediation of industrial effluents with variable composition of inhibitors^30^.

Synthetic dyes are extensively used in different industries, and 10–15% of dyes used during finishing and dyeing processes ends up in wastewater^31^. Anthraquinone-based reactive dyes have attracted attention from the environmental and toxicological perspective, since they are extremely resistant to degradation and present acute toxicity of mutagenic effects^31^. Conventional biological systems are not efficient for bioremediation and decolorization of dyes, and physico-chemical methods are associated to high operational cost, formation of undesirable by-products, etc. NS-CotA can be an eco-friendlier and practical choice for the bioremediation of dye polluted wastewater effluents. In this sense, the NS-contained enzyme was shown to decolorate the anthraquinonic dye RB19 quite efficiently compared to free enzyme. Although the RB19 decolorization rate was lower than that recently reported by using *Trametes versicolor* laccase in solution^32^, it was remarkably higher than that achieved by an immobilized fungal laccase in absence of a mediator^33^ (only 5% decolorization in 6 h). Furthermore, it should be highlighted that the transformation of the dye by NS-laccase could be optimized in terms of pH, enzyme dosage or temperature, for a more efficient transformation of the substrate.

Also, because of their particulate nature, the NS-contained enzyme can be recovered from the reaction and re-used for several times without a significant loss in activity. Furthermore, because their small size, some NS might have been lost during the repeated centrifugation steps and the small decay in activity can be probably explained by diminished protein content. An improved recovery method, involving filtration, NS-linking, or others, can be implemented to improve the recovery rates.

The most remarkable property that we have observed for this system is the thermal stabilization achieved. It is certain that the *B. subtilis* laccase is already intrinsically heat-stable. However, the NS-contained version surpasses extensively the abilities of the free enzyme. Although the encapsulated version outperforms the free one over all the temperature range, is at the highest values of 90 and 95 °C where the differences become remarkable. Thus, while the activity of the free enzyme is completely abrogated after 5 min incubation at 90 °C, the NS-contained laccase resisted at least 30 min of incubation at that temperature without any appreciable activity loss. The performance of the NS-laccase only started to decline to a 77% of the initial activity after 15 min of incubation at 95 °C. This extreme thermostability might be beneficial for transformations that might be accelerated by increasing the temperature, well laccase-mediated oxidations or multi-enzymatic complex reactions.

The IC-tagging methodology has been previously shown to produce micro and nano-encapsulated properly folded proteins. Here we have shown that this simple methodology can be also utilized for the production of active enzymes for industrial or other different uses, and that enzymes get stabilized while integrated into the NS and can be easily recovered and further reused. Between the IC-tagging characteristics are its simplicity, versatility, and the fact that it allows us to express and immobilize proteins and enzymes in one step, via production in either prokaryotic or eukaryotic cells. Once produced, the purification technique of these MS and NS is certainly straightforward, consisting in the use of cell lysis and centrifugation cycles bypassing the use of purification columns and other expensive methods, making this platform inexpensive and fewer time consuming. Also, we have previously demonstrated that several proteins can be simultaneously integrated into the same MS or NS, making them suitable for the easy creation of micro/nano reactors that integrate different immobilized enzymes for connected funneled multi-step transformations^34,35^. The stabilization observed can be particularly useful when performing complex reactions that require the use of different enzymatic activities requiring different co-factors that may not be fully compatible with all the participating enzymes.

## Supplementary Information

Supplementary Information
