# Supplementary material for: Chemical and thermal stabilization of CotA laccase via a novel one-step expression and immobilization in muNS-Mi nanospheres
Source: Sci Rep. 2021 Feb 2;11:2802. doi: 10.1038/s41598-021-82468-x (PMC7854631; doi:10.1038/s41598-021-82468-x)
Supplement: Supplementary file 1 — Supplementary Information [file 41598_2021_82468_MOESM1_ESM.pdf]

Supplementary Information

Chemical and thermal stabilization of CotA laccase via a novel one-step expression and immobilization in muNS-Mi nanospheres

Authors: *Pose-Boirazian, Tomás;[1] Eibes, Gemma;[2] Barreiro-Piñeiro, Natalia;[1] Díaz Jullien, Cristina;[3] Lema, Juan M.;[2] Martínez-Costas, Jose.[1,]**

1-Centro Singular de Investigación en Química Biolóxica e Materiais Moleculares (CiQUS), Departamento de Bioquímica y Biología Molecular, Universidade de Santiago de Compostela, 15782 Santiago de Compostela, Spain.

2- CRETUS Institute, Dept. of Chemical Engineering, Universidade de Santiago de Compostela, E-15782 Santiago de Compostela, Spain.

3- Centro de Investigación en Bioloxía (CiBUS), Departamento de Bioquímica y Biología Molecular, Universidade de Santiago de Compostela, 15782 Santiago de Compostela, Spain.

Correspondence to: jose.martinez.costas@usc.es

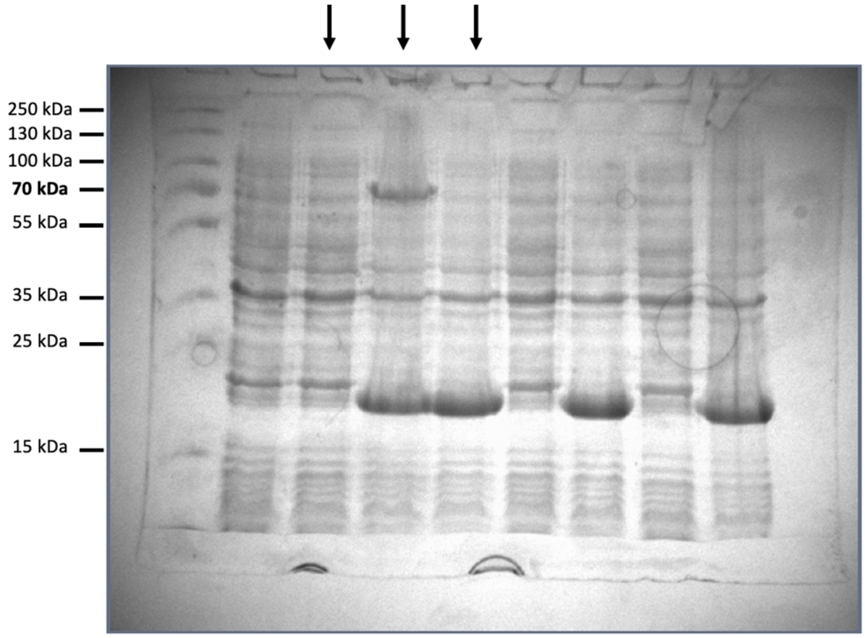

Figure S1- Full Coomassie-stained gel containing (arrows) the lanes shown in Fig. 1a on the main manuscript. The the weights corresponding to the markers included in the first lane of the gel are indicated at the left.

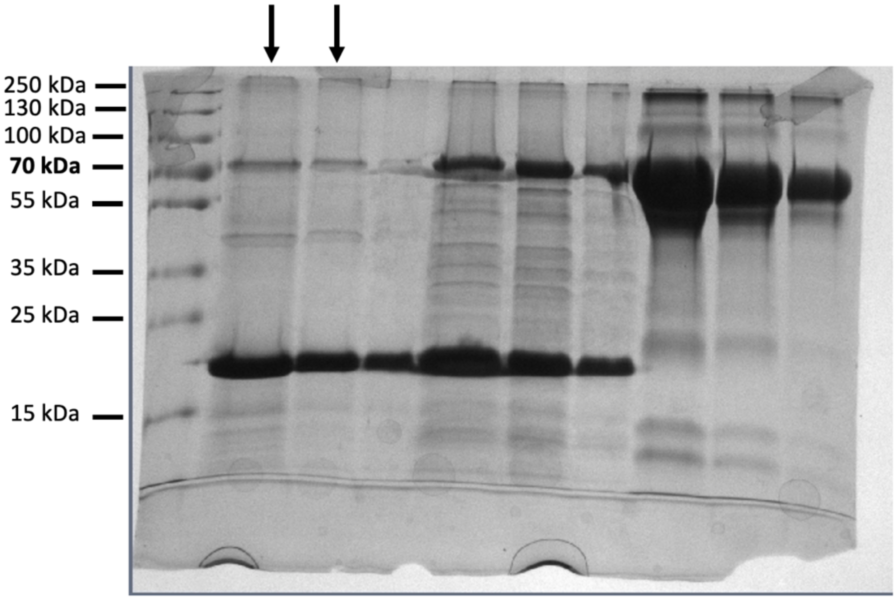

Figure S2- Full Coomassie-stained gel containing (arrows) the lanes shown in Fig. 1b on the main manuscript. The the weights corresponding to the markers included in the first lane of the gel are indicated at the left.
